# Supplementary material for: Causal Relationship between Aging and Anorexia Nervosa: A White-Matter-Microstructure-Mediated Mendelian Randomization Analysis
Source: Biomedicines. 2024 Aug 16;12(8):1874. doi: 10.3390/biomedicines12081874 (PMC11351342; doi:10.3390/biomedicines12081874)
Supplement: Supplementary file 1 [file biomedicines-12-01874-s001.zip › Supplementary Figure/Figure S8/Leaveoneout plot of MR analysis for TL to ubm-b-1680.pdf]

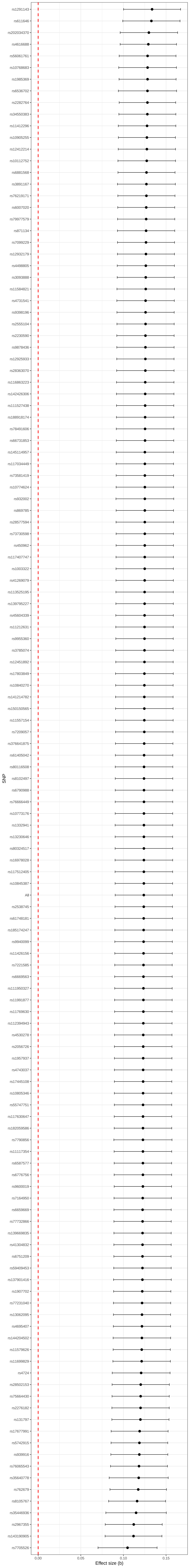
rs1291143
rs611646
rs202034370
rs4616688
rs56061761
rs10768683
rs1985369
rs6536702
rs2282764
rs34550383
rs11412296
rs10905255
rs12412214
rs10112752
rs6881568
rs3891167
rs76219171
rs6007020
rs79977579
rs871134
rs7099229
rs12932179
rs4498805
rs3093888
rs11584821
rs4731541
rs9398196
rs2555104
rs2230590
rs9878436
rs12925933
rs28363070
rs116863223
rs142426306
rs111527438
rs188918174
rs78491606
rs66731853
rs145114957
rs117034449
rs73581419
rs10774624
rs932002
rs869785
rs28577594
rs73730598
rs450962
rs117407747
rs1003322
rs41269079
rs113525195
rs139795227
rs45604339
rs11212631
rs9955360
rs3785074
rs12451892
rs17803849
rs10840270
rs141214782
rs150150565
rs11557154
rs7209057
rs376641875
rs61405042
rs80116508
rs8102497
rs6790988
rs76666449
rs10773176
rs1332941
rs13230646
rs80324517
rs16978028
rs117512405
rs10845387
All
rs2538745
rs61748181
rs185174247
rs9940099
rs11426156
rs7221585
rs6669563
rs111950327
rs11991877
rs11769630
rs112394943
rs4530278
rs2056726
rs1957937
rs4743037
rs17445108
rs10805346
rs55747751
rs117630647
rs182059586
rs7790856
rs11117354
rs6587577
rs6776756
rs9600019
rs7164950
rs6659669
rs77732866
rs139669835
rs41304832
rs6751209
rs59409453
rs137901416
rs1907702
rs77231040
rs13062095
rs4695407
rs144204502
rs11579626
rs11699829
rs4724
rs28502153
rs75664430
rs2276182
rs131797
rs17677991
rs5742915
rs939916
rs76065543
rs35640778
rs762679
rs8105767
rs35446936
rs2967355
rs143190905
rs7705526
SNP
0.00
0.05
0.10
0.15
Effect size (b)
